# YouTube as an information source in deep margin elevation: Reliability, accuracy and quality analysis

**DOI:** 10.1371/journal.pone.0318568

**Published:** 2025-02-07

**Authors:** Zeyneb Merve Ozdemir, Sevim Atılan Yavuz, Derya Gursel Surmelioglu

**Affiliations:** 1 Department of Restorative Dentistry, Faculty of Dentistry, Kahramanmaras Sutcu Imam University, Onikisubat, Kahramanmaras, Turkey; 2 Department of Restorative Dentistry, Faculty of Dentistry, Mersin University, Mersin, Turkey; 3 Department of Restorative Dentistry, Faculty of Dentistry, Gaziantep University, Sehitkamil, Gaziantep, Turkey; University of Sharjah College of Dental Medicine, UNITED ARAB EMIRATES

## Abstract

The objective of this research was to assess the accuracy, quality, content, and demographics of videos on YouTube concerning deep margin elevation (DME). Initially, 100 videos for each of the three keywords were analyzed. The content categories of these videos were diverse, encompassing educational materials, teaching techniques, advertisements, and other types of content. The evaluation of the videos was carried out based on the Global Quality Scale (GQS), the Journal of the American Medical Association (JAMA) benchmark, and the modified-DISCERN questionnaire (m-DISCERN). Non-distributed data were analyzed using the Kruskal Wallis test and the Spearman correlation coefficient. The JAMA score was 1 for four videos, 2–3 for 38, and 4 for 14 videos; the GQS score was 1–2 for 18 videos, 3 for 11 videos, and 4–5 for 27 videos; and the m-DISCERN score was < 3 for 39 videos, 3 for four videos, and > 3 for 13 (for a total of 56 videos). Statistically significant differences were observed only for the JAMA scores when comparing the video source groups (p = 0.001). There were significant positive correlations between the GQS and m-DISCERN and m-DISCERN and JAMA scores (p < 0.001 and p = 0.049, respectively). The findings indicated that YouTube videos related to DME generally exhibited high-quality content but only moderate accuracy and poor reliability.

## Introduction

In clinical practice, managing teeth with subgingival defects can present a considerable challenge. Restoring damaged teeth can be an arduous task due to the difficulty of maintaining optimal moisture and contamination control during the process. Various treatment options exist for retaining and restoring teeth with subgingival defects. With each option presenting its own set of pros and cons, it is crucial to carefully evaluate the specific circumstances of each case in order to determine the most suitable choice, taking into consideration both clinical factors and the needs and expectations of the patient. Surgical extrusion is invasive and risky, and while orthodontic forced eruption preserves the pulp, it is time consuming; as another alternative, surgical crown lengthening is costly, time consuming, and uncomfortable for patients [1]. One other more conservative method, referred to as deep margin elevation (DME), involves applying a resin-based composite layer over the cervical margin to elevate it coronally [2, 3]. The technique, initially presented by Dietschi and Spreafico in 1998 [3], is alternatively referred to as "proximal box elevation" or "cervical margin relocation". This approach offers several advantages, including precise isolation with the aid of a rubber dam, moisture management, straightforward impression taking, appropriate bonding techniques, and the elimination of excess material while avoiding unnecessary tissue sacrifice [2, 4]. The open-sandwich technique is generally recognized as a precursor of DME [5]. Despite the apparent similarities between the two approaches, DME was originally introduced for indirect restorations employing resin-based composite restoration [3]. Performing DME reduces the thickness of indirect restorations and improves the curing process. It also preserves sound tooth substances by allowing for direct restoration in specific areas. Likewise, DME can facilitate the adhesion of ceramics in deep proximal regions [1, 6, 7].

Social media has revolutionized the way people present their own ideas and other information to others. Today, individuals have the freedom to choose the format that best suits their needs and to create content that is easily accessible. With widespread access to websites and social media platforms, an extensive number of individuals around the world now have access to daily healthcare updates [8]. Traditionally, medical and dental information has been obtained through direct consultation with specialists. However, in modern times, the widespread use of the internet in developed countries has led to a growing trend of individuals seeking this information online [9]. The information disseminated through social media platforms with regard to health is not limited solely to informing patients, as it is also employed as an educational tool for students. Dentists and medical professionals commonly utilize social media platforms, such as Wikipedia, YouTube, and Facebook, to access information using diverse educational approaches [10, 11].

The website commonly known as YouTube was established in June 2005 as an extensive online platform for user-generated content [12]. In the domain of dental and medical education, a significant body of research has revealed that YouTube holds the distinction of being the most commonly utilized electronic resource among students pursuing careers in healthcare, and it is widely regarded as an instrument for acquiring practical knowledge in a variety of disciplines [13]. The utilization of visual demonstrations, such as those provided by YouTube videos, serves as a form of e-learning [13]. Interestingly, though, among the 40 most searched dental videos on YouTube, dental schools direct students to only 5% of this content [14]. While video-based lectures are not universally included in dental curricula [15], student test results have been found to be significantly higher when videos are used for instructional purposes compared to textbooks alone [16]. One particular study on dental students reported that 95% found YouTube videos helpful for learning clinical procedures, and 89% desired similar tutorials to be posted by their schools on YouTube and social media [17]. Nevertheless, the reliability, accuracy, and scientific validity of the content presented in YouTube videos may be questionable, leading to the dissemination of unclear, incorrect, and misleading information. Furthermore, a significant portion of the electronic learning resources accessible through the internet may not have been created by licensed dental professionals or educators [18, 19]. Video-based learning tools can be efficacious for enhancing the education of dental students, particularly in visual processes requiring precision, such as DME. Accordingly, the objective of the present investigation was to assess the video content related to DME available on YouTube with regard to the accuracy of the information and the educational level of the content.

The null hypotheses consisted of the following; (i) there are no differences in JAMA, m-DISCERN, and GQS scores across source categories; (ii) there are no differences in JAMA, m-DISCERN, and GQS scores across content categories; and (iii) there is no significant relationship between video variables and JAMA, DISCERN, and GQS scores

## Materials and methods

The present investigation consisted of a cross-sectional study that integrated both qualitative and quantitative analyses of video content available on YouTube, a platform owned by Alphabet Inc. (Mountain View, CA, USA), with a particular focus on the topic of the DME. This research did not involve any clinical data, human participants, or animal subjects. The study relied solely on publicly available YouTube videos, without accessing any personal information. As a result, there was no direct interaction with users, thereby eliminating the need for an ethical review process.

### Video selection

The process of screening eligible videos was carried out within a time frame of three days, taking into consideration the significant volume of videos that are generated and uploaded on a daily basis, as well as the rapidly evolving nature of social media. A primary investigation was conducted by a solitary researcher (OZM) with the employment of the keywords “deep margin elevation,” “cervical margin relocation,” and “proximal box elevation” on the YouTube search engine (https://www.youtube.com/) on December 29, 2023. The investigation was conducted by establishing a new, unused YouTube account. This approach was applied because the YouTube algorithm takes into account user interactions when recommending content [14]. To commence the evaluation process, the initial 100 videos identified for each keyword were meticulously assessed. To analyze and evaluate the videos, the researchers dedicated approximately two weeks.

### Exclusion criteria

The following exclusion criteria were applied to eliminate videos from the analysis: (1) those utilizing a language other than English, (2) duplicate videos, (3) those lacking both audio and visual content, (4) videos not directly relevant to DME, (5) videos longer than 30 minutes in duration, and (6) YouTube shorts.

### Inclusion criteria

The selection criteria for the videos were as follows: the video had to (1) demonstrate a teaching technique and/or procedures related to DME and (2) be uploaded in English on YouTube. To determine the target audience of each video, the speech and language were evaluated. The researchers also considered videos that documented the procedures performed on the patients.

### Video metrics analysis

The evaluation of videos was conducted by assessing various parameters, such as (1) the number of views, (2) the number of likes, (3) the number of dislikes, (4) the number of comments, (5) video duration (seconds), (6) days since upload, (7) viewing rate, (8) interaction index (II), and (9) the video power index (VPI). The video uploaders were categorized into six categories, which consisted of (1) dental professionals, (2) specialists, (3) dental companies, (4) health-related institutions, (5) YouTube channels, and (6) other. The videos were classified under four headings based on their content: (1) educational, (2) teaching technique, (3) advertisement, and (4) other. Although the display of dislike numbers has been discontinued following a recent YouTube update, it is still possible to measure both the II and VPI using the Return YouTube Dislike extension. This extension can be downloaded and accessed through Google Chrome.

In the initial analysis, various video metrics were taken into account, including video duration; the number of days since its upload; the total number of views, likes, and dislikes; and the number of comments. These metrics, namely VPI, viewing rate, and II, were calculated as measures of video popularity.

The formula used to calculate the VPI, viewing rate, and II were as follows [20, 21]:

VPI: [like rate * view rate / 100]. Like rate is calculated with formula [likes * 100 / (likes + dislikes)], and view rate is calculated with (number of views / days)

Viewing Rate (%): (number of views / number of days since upload) * 100

II (%): [(number of likes- number of dislikes) / (total number of views)] * 100

### Assessment of video accuracy, quality and reliability

The evaluation process for the 300 videos involved assessing their ability to provide useful information to viewers and determining their reliability and accuracy. To this end, several scales were utilized, including the Global Quality Score (GQS), the Journal of the American Medical Association (JAMA) Benchmark Criteria, and the modified DISCERN questionnaire. The sufficiency of information related to DME was assessed by evaluating the videos’ performance based on these criteria. The authors rated the videos and analyzed the results according to the GQS, the m-DISCERN, and the JAMA scores. In cases of disagreement regarding the scores, the authors worked to reach a consensus at the end of the scoring process. Each author performed the evaluation individually, and their scores were compared with the results of a kappa test. A kappa statistic ≤ 0 indicated “poor agreement,” > 0 but ≤ 0.20 denoted “slight agreement,” 0.21–0.40 signified “fair agreement,” 0.41–0.60 represented “moderate agreement,” 0.61–0.80 was indicative of “substantial agreement,” and > 0.80 indicated “almost perfect agreement” [22].

**Fig 1:**
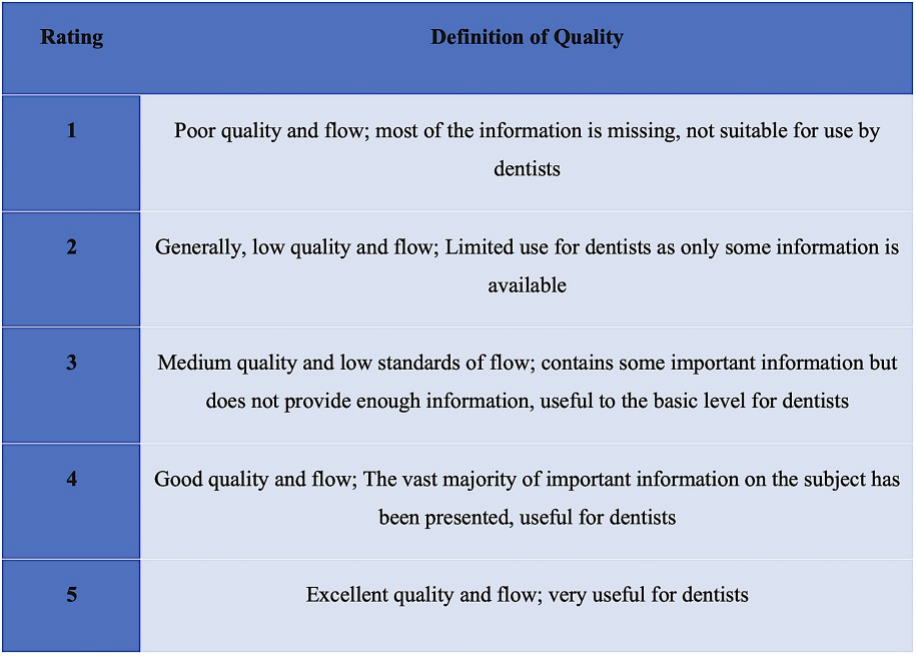
Global Quality Score (GQS) scoring system.

The GQS was employed to evaluate the content and structure of the videos, with a range of 1 to 5 points, where 1 signified low quality, and 5 indicated high quality. The evaluation concentrated on the overall quality of the videos, taking into account their coherence, flow, and the accuracy of the information they presented. The content quality scale developed by Bernard et al. [23] was used to assess the level of information accessibility and flow on websites, with a score of 1–2 indicating low content quality, 3 indicating intermediate content quality, and 4–5 indicating high content quality. A higher score on this scale reflected a greater quality of information (Fig 1).

**Fig 2:**
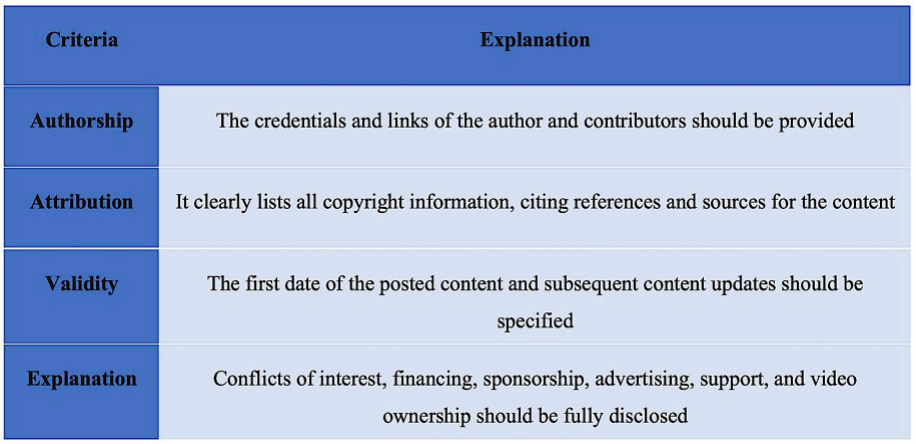
The Journal of American Medical Association (JAMA) comparison criteria.

The JAMA benchmark criteria is a widely recognized four-point scale utilized to assess the reliability and accuracy of videos and resources. This scale evaluates materials according to four key criteria: authorship, attribution, disclosure, and currency (Fig 2). Each criterion is allocated one point by the examiner, resulting in a total score ranging from 0 to 4 points. A score of one point signifies a low level of accuracy, while scores of two to three points indicate a partially accurate medium level, and a score of four points signifies a high level of source accuracy [24].

**Fig 3:**
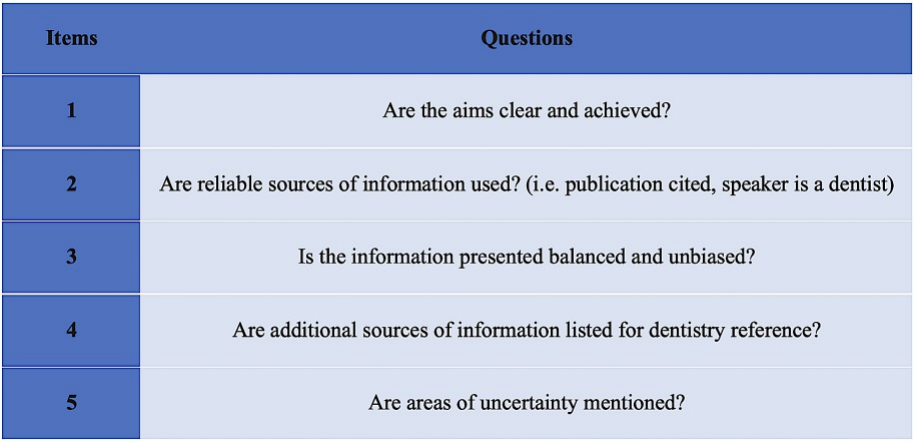
Modified DISCERN questionnaire.

The m-DISCERN questionnaire is a tool that comprises five items designed to evaluate the reliability of resources by assessing specific features. The tool features five yes/no questions that examine the resources’ reliability (Fig 3). The total score is determined by adding up the “yes” responses, each of which is worth one point. A score of 0 indicates the least dependable resource reliability, while a score of 5 signifies the highest resource reliability. These inquiries assess the dependability of the resources by evaluating their attributes. Per the assessment of the m-DISCERN questionnaire, video scores surpassing 3 points signify a good level of reliability, a score of 3 points denotes a moderate level of reliability, and scores below 3 points suggest a poor level of reliability [25].

### Statistical analysis

The data analysis was conducted using SPSS version 26 for Windows (Chicago, IL, USA). The normality of the data was determined using the Shapiro–Wilk test, which indicated that the data did not adhere to a normal distribution. Consequently, the Kruskal–Wallis and Dunnett’s post hoc tests were employed to compare JAMA, GQS, and m-DISCERN scores between sources and content groups. The kappa statistic was calculated to assess interrater reliability for the JAMA, GQS, and m-DISCERN scores’ classifications. To examine the potential correlation between the JAMA, GQS, and m-DISCERN scores, Spearman’s correlation coefficient was calculated. The level of statistical significance was set at p = 0.05.

## Results

**Fig 4:**
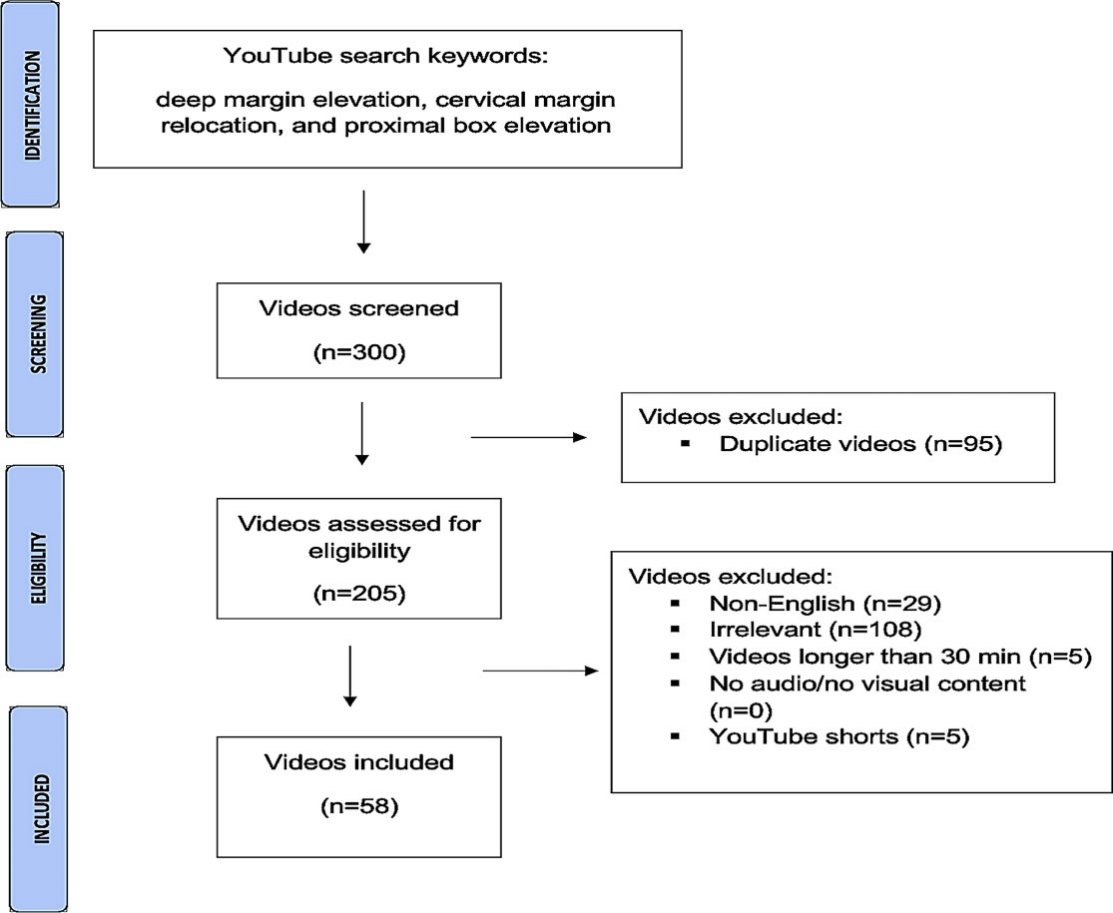
Flow chart of the video selections.

The initial screening process involved a total of 300 videos, from which 58 were ultimately selected for inclusion in the study following the application of the eligibility criteria (Fig 4).

After eliminating duplicates from a collection of 300 videos, 58 videos were selected for evaluation. Unfortunately, two of these videos were deemed ineligible for inclusion due to the source not granting permission. Therefore, 56 videos were ultimately analyzed. The analysis of the evaluated video data revealed that a total of 25923 seconds were examined, and the average length of these videos was calculated to be 462.91 seconds. Furthermore, it was determined that the videos received a total of 15772 likes, with an average of 281.64 likes per video. On the other hand, a total of 512 dislikes were recorded, resulting in an average of 9.14 dislikes per video. According to Cohen’s kappa statistic, the interrater reliability between the authors was 0.941 for the GQS, 0.931 for the JAMA, and 0.900 for the m-DISCERN.

### Outcomes for sources

**Fig 5:**
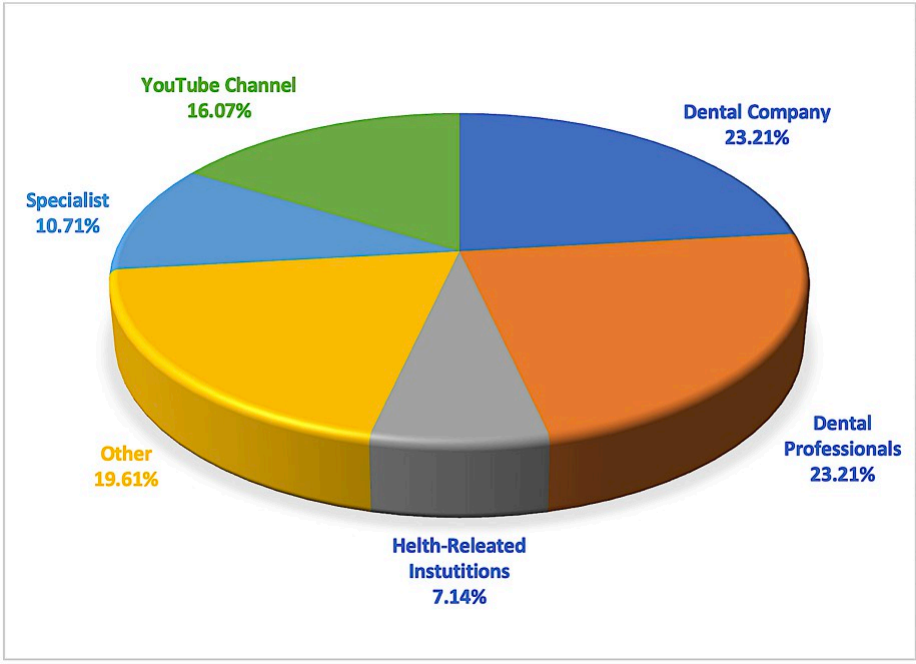
Categorization of YouTube videos based on the sources.

Based on the results of the video source classification, it was found that dental professionals and dental companies accounted for the largest proportion of accounts among all categories, with a total of 23.21% (n = 13) falling into this classification. The findings revealed that the health-related institutions constituted the lowest proportion of uploaded video sources, accounting for 7.14% of the total number of sources (n = 4). Additionally, “other” sources accounted for 19.61%, YouTube channels comprised 16.07%, and specialists represented 10.71%. A representation of the distribution of the video sources is depicted in Fig 5. It was only with respect to the JAMA criteria that there was a significant difference among the sources (p < 0.001), whereas for the GQS and m-DISCERN scores, no significant differences were found between the uploaded sources (p > 0.050). In terms of the JAMA scores for the sources, there were significant differences between dental companies and dental professionals, specialists, and other sources (p < 0.001). This is particularly attributable to the fact that these uploaders provide more accurate information during the upload process and share more comprehensive details about the channel to which they are uploading. In terms of the GQS, the mean value of videos uploaded by the dental company was 3.46, dental professionals were 3.77, health-related institutions were 4.25, other was 3.55, specialists were 2.33, and YouTube channels was 2.33. While there were generally high-quality videos, it is noteworthy that specialists were more likely to upload low-quality videos than the others. In terms of the JAMA, the mean value of videos uploaded by dental companies was 3.62, dental professionals were 2.31, health-related institutions were 3.25, other was 1.73, specialists were 2.00, and YouTube channels was 2.44. It has been observed that videos uploaded by professionals (dental professionals/specialists) generally have only partially medium accuracy. Finally, in terms of m-DISCERN, the mean value of the videos uploaded by dental companies was 2.62, dental professionals were 2.38, health-related institutions were 3.00, other was 2.27, specialists were 2.67, and YouTube channels was 1.89. A comparison of assessment scores based on sources is shown in Table 1.

**Table 1:** Comparison of assessment scores based on sources represented as median (first quartile-third quartile) values.

*Variable*	*Dental Professionals (n = 13)*	*Specialist (n = 6)*	*Dental Company (n = 13)*	*Health-Related Institutions (n = 4)*	*YouTube Channel (n = 9)*	*Other (n = 11)*	*p value*
***Number of views***	5721 (990–32249.5)	3228 (1073.5–46315.75)	4109 (2066.5–10787.5)	24886.5 (16483.25–49272.25)	891 (131–5040)	2639 (873–20999)	0.779
***Number of likes***	90 (33–628.5)	66 (12.75–723.75)	61 (28.5–216.5)	539 (285.5–1242.5)	7 (4.5–38.5)	42 (11–259)	0.341
***Number of dislikes***	0 (0–12)	0 (0–9.75)	1 (0–4)	13.5 (5.75–26.5)	0 (0–1.5)	2 (0–6)	0.772
***Number of comments***	8 (3–34)	7.5 (2.25–38.5)	5 (0.5–5)	10 (1.25–69.75)	1 (1–2.5)	3 (0–7)	0.406
***Video duration (sec)***	329 (109.5–634.5)	555.5 (113.25–900)	251 (109–838)	978.5 (373.25–1463.75)	227 (108–458.5)	215 (76–623)	0.232
***Days since upload***	922 (276.5–1885)	381.5 (188–912)	476 (400.5–916)	1026 (635–2974.75)	1045 (429–1999.5)	974 (583–1823)	0.198
***Interaction index (II)***	1.72 (1.325–4.52)	1.47 (0.8925–2.9425)	1.62 (1.125–1.8)	2.28 (1.4125–2.6725)	0.93 (0.585–3.195)	1.65 (1.26–2.12)	0.147
***Viewing rate***	1495.58 (264.4–3639.74)	920.68 (119.44–6130.0975)	1305.95 (350.43–2521.09)	2915.47 (1103.54–4249.685)	139 (44.39–348.71)	202.53 (110.36–928.16)	0.364
***VPI***	14.56 (2.585–35.93)	9.21 (1.1625–60.46)	13.06 (3.505–25.035)	28.35 (10.805–41.74)	1.39 (0.445–3.385)	1.96 (0.95–8.91)	0.333
***GQS***	5 (2.5–5)	2.5 (1.75–4.25)	4 (2.5–5)	5 (2.75–5)	2 (1.5–3)	4 (2–5)	0.135
***JAMA***	2 (2–2.5)^A^	2 (2–2)^A^	4 (3–4)^B^	3.5 (2.25–4)^AB^	2 (1–2)^AB^	2 (1–2)^A^	< 0.001*
***Modified DISCERN***	2 (1.5–3)	2.5 (1.5–4.25)	2 (1.5–3.5)	3 (1.25–4.75)	2 (2–3)	2 (1–2)	0.791

*GQS: Global Quality Scale, JAMA: Journal of American Medical Association benchmark criteria

** Upper cases indicate significant differences between lines. Values are presented as median (first quartile-third quartile)

### Outcomes for contents

The GQS results had a median value of 3.00, whereas both the JAMA and m-DISCERN had a median value of 2.00. Table 2 displays the descriptive data for the videos. The videos had mostly a high content quality (4–5 points; 48%, n = 27). However, in terms of the JAMA and the m-DISCERN, the videos mainly had a medium accuracy level (2–3 points; 68%, n = 38) and poor reliability (< 3 points; 70%, n = 39). Overall, it was observed that the majority of the videos exhibited high content quality, with medium accuracy and poor reliability. Assessments of the parameters of the videos are shown in Table 3.

**Table 2:** Descriptive data of the videos represented as median (first quartile-third quartile).

*Data*	*Value (n = 56)*
***Number of views***	3609 (1000.25–15665.5)
***Number of likes***	54 (11.25–273.25)
***Number of dislikes***	0 (0–5.75)
***Number of comments***	3 (0–12.5)
***Video duration (Sec)***	301 (122–690)
***Days since upload***	790.5 (463.75–1533.75)
***Interaction index (II)***	1.63 (1.1125–2.5225)
***Viewing rate***	524.23 (157.4575–2671.3625)
***VPI***	5.18 (1.575–24.1425)
***GQS***	3 (2–5)
***JAMA***	2 (2–3.75)
***Modified DISCERN***	2 (1–3)

*Values are presented as median (first quartile-third quartile)

**GQS: Global Quality Scale, JAMA: Journal of American Medical Association benchmark criteria

**Table 3:** Assessment of parameters of the videos represented as N (%).

*Parameter*	*Value (n = 56)*
**GQS (1–5 points)**	
***Low Content Quality (1–2 points)***	18 (32%)
***Intermediate Content Quality (3 points)***	11 (20%)
***High Content Quality (4–5 points)***	27 (48%)
***JAMA Score (0–4 points)***	
***Low Level Accuracy (1 point)***	4 (7%)
***Partially Medium Accuracy (2–3 points)***	38 (68%)
***High Level Accuracy (4 points)***	14 (25%)
***Modified DISCERN Score (0–5 points)***	
***Poor Reliability (<3 points)***	39 (70%)
***Moderate Reliability (3 points)***	4 (7%)
***Good Reliability (>3 points)***	13 (23%)

*Values are presented as number (%).

**GQS: Global Quality Scale, JAMA: Journal of American Medical Association benchmark criteria

When the videos were compared in terms of content (teaching technique, educational, advertisement and other) significant differences were found only concerning the GQS, the JAMA criteria and the m-DISCERN parameters, but not the descriptive data (p = 0.015, p < 0.001, p < 0.001, respectively). In terms of the “teaching technique” category, the mean quality of the videos was 3.45, the mean reliability was 1.81, and the mean accuracy was 2.13. On the other hand, for the “education” category, the mean quality of videos was 3.74, the mean reliability was 3.79, and the mean accuracy was 3.18. In terms of “advertisements,” the mean quality was 1.50, the mean reliability was 1.00, and the mean accuracy was 3.25. Finally, with respect to the “other” category, the mean quality was 2.00, the mean reliability was 1.50, and the mean accuracy was 2.00. The values of the videos according to content are shown in Table 4.

**Table 4:** Values of videos according to content represented as median (first quartile-third quartile).

*Variable*	*Teaching Technique (n = 31)*	*Educational (n = 19)*	*Advertisement (n = 4)*	*Other (n = 2)*	*p value*
***GQS***	3 (2–5)^A^	4 (3–5)^A^	1.5 (1–2)^B^	2 (1–3)^AB^	0.015
***Modified DISCERN***	2 (1–2)^A^	4 (3–5)^B^	1 (1–1)^C^	1.5 (1–2)^ABC^	< 0.001
***JAMA***	2 (2–2)^A^	4 (2–4)^B^	3.5 (2.25–4)^AB^	2 (2–2)^A^	< 0.001

*GQS: Global Quality Scale, JAMA: Journal of American Medical Association benchmark criteria

** Upper cases indicate significant differences between lines.

***Values are presented as median (first quartile-third quartile)

### Outcomes regarding correlations between accuracy, content of quality, and reliability

The results indicated a substantial positive correlation between GQS and the number of views (r = 0.418, p < 0.001), number of likes (r = 0.425, p = 0.001), number of dislikes (r = 0.466, p < 0.001), number of comments (r = 0.224, p = 0.048), video duration (r = 0.683, p < 0.001), viewing rate (r = 0.405, p = 0.001), and VPI (r = 0.401, p = 0.001). As the duration of the videos increased, both quality and reliability increased, as the information provided in the videos increased. In addition, as the quality of the videos increased, the number of viewers and likes of the videos increased; thus, the number of dislikes and comments increased as the popularity of the video increased. This resulted in an increase in the viewing rate and the VPI.

There was a positive correlation between the JAMA benchmark results and the number of views (r = 0.238, p = 0.039), viewing rate (r = 0.247, p = 0.033), and VPI (r = 0.255, p = 0.029). Similarly, there was a positive correlation between the m-DISCERN scores and the number of dislikes (r = 0.233, p = 0.049) and video duration (r = 0.575, p < 0.001). A positive correlation was also found between m-DISCERN–GQS (r = 0.610, p < 0.001) and m-DISCERN–JAMA (r = 0.223, p = 0.049). Table 5 displays the correlations between the variables and scores.

**Table 5:** Correlations between quantitative variables and scores.

*Variable*	*GQS*	*JAMA*	*Modified DISCERN*
***Number of views***	r = 0.418 p = 0.001	r = 0.238 p = 0.039	r = 0.125
***Number of likes***	r = 0.425 p = 0.001	r = 0.220	r = 0.161
***Number of dislikes***	r = 0.466 p < 0.001	r = 0.161	r = 0.223 p = 0.049
***Number of comments***	r = 0.224 p = 0.048	r = -0.049	r = 0.050
***Video duration (sec)***	r = 0.683 p < 0.001	r = 0.156	r = 0.575 p < 0.001
***Days since upload***	r = 0.193	r = -0.025	r = 0.095
***Interaction index (II)***	r = 0.104	r = -0.025	r = 0.201
***Viewing rate***	r = 0.405 p = 0.001	r = 0.247 p = 0.033	r = 0.077
***VPI***	r = 0.401 p = 0.001	r = 0.255 p = 0.029	r = 0.075
***GQS***	-	r = 0.190	r = 0.610 p < 0.001
***JAMA***	r = 0.190	-	r = 0.223 p = 0.049
***Modified DISCERN***	r = 0.610 p < 0.001	r = 0.223 p = 0.049	-

*Nonparametric Spearman’s rank correlation coefficients (rs) are displayed.

**GQS: Global Quality Score, JAMA: Journal of American Medical Association benchmark criteria.

### Outcomes for accuracy, content of quality, and reliability

The video metric data obtained were analyzed using the GQS, the JAMA, and m-DISCERN; the JAMA criteria were evaluated according to accuracy level (low level, medium, and high level) for each video metric and criterion, and no significance difference was found (p > 0.050). However, there were statistically significant differences between m-DISCERN scores (poor reliability, moderate reliability, and good reliability) and video duration, the GQS, and the JAMA criteria (p = 0.002, p = 0.016, and p = 0.002, respectively). In terms of the GQS scale (low content, intermediate content, and high content), statistically significant differences were found between GQS and the number of likes, number of comments, video duration, viewing rate, VPI, and -m-DISCERN scores (p = 0.003, p = 0.027, p = 0.001, p = 0.028, p = 0.025, and p = 0.001, respectively). The video duration of videos with good reliability was significantly longer than that of those with moderate and poor reliability. Furthermore, the accuracy of videos with good and moderate reliability was similar. Similarly, videos with good reliability were of higher quality than those with poor reliability. These outcomes show that long videos were more reliable, and videos with good reliability were of higher quality and accuracy. In addition, the likes, comments, video duration, viewing rate, VPI, and reliability of videos with high quality content were significantly higher than those with low or intermediate quality content. As a result, it has been observed that high-quality videos show more interaction, receive more likes and comments, have a higher viewing rate, and are more reliable. Video metric data related to the m-DISCERN in Table 6 and data related to the GQS scale are shown in Table 7.

**Table 6:** Modified DISCERN values represented as median (first quartile-third quartile).

*Variable*	*Poor (n = 39)*	*Moderate (n = 4)*	*Good (n = 13)*	*p value*
***Number of views***	2639 (891–14372)	4664 (1813–12618)	8597 (1862–39764)	0.184
***Number of likes***	42 (11–211)	87.5 (33.25–232.5)	259 (10–956)	0.203
***Number of dislikes***	0 (0–5)	1 (0–2)	5 (1–14)	0.105
***Number of comments***	3 (1–8)	4 (1.5–6.5)	7 (0.5–41)	0.358
***Video duration (sec)***	202 (81–434)^A^	336.5 (146.75–430.25)^A^	723 (500–1076)^B^	0.002*
***Days since upload***	775 (455–1344)	766 (470.75–1064.25)	923 (465.5–2477)	0.286
***Interaction index (II)***	1.45 (1.09–2.12)	1.8 (1.3–2.57)	2.44 (0.725–3.375)	0.701
***Viewing rate***	423.64 (165.34–2045.66)	854.23 (171.1125–2656.315)	642.55 (71.88–4086.015)	0.505
***VPI***	3.93 (1.65–20.34)	8.47 (1.6775–26.385)	6.25 (0.64–40.33)	0.567
***GQS***	3 (2–4)^A^	4 (2.5–4.75)^AB^	5 (4–5)^B^	0.016*
***JAMA***	2 (2–3)^A^	4 (2.5–4)^B^	3 (2–4)^B^	0.002*

*GQS: Global Quality Score, JAMA: Journal of American Medical Association benchmark criteria

** Upper cases indicate significant differences between lines.

***Values are presented as median (first quartile-third quartile)

**Table 7:** GQS values represented as median (first quartile-third quartile).

*Variable*	*Low content quality (n = 18)*	*Intermediate content quality (n = 11)*	*High content quality (n = 27)*	*p value*
***Number of views***	2507 (886.5–4494)	1786 (245–7257)	14497 (2151–33735)	0.181
***Number of likes***	38.5 (10.75–109)^A^	31 (5–63)^A^	255 (29–770)^B^	0.003*
***Number of dislikes***	0 (0–1.25)	0 (0–5)	4 (0–15)	0.194
***Number of comments***	3 (1–7.25)^A^	2 (0–6)^A^	5 (1–36)^B^	0.027*
***Video duration (sec)***	90.5 (67.75–231.25)^A^	290 (139–517)^A^	623 (271–1058)^B^	0.001*
***Days since upload***	553 (217.75–1117.75)	775 (462–1713)	923 (476–1823)	0.165
***Interaction index (II)***	1.49 (1.0975–2.5775)	1.57 (0.61–2.04)	1.72 (1.25–2.75)	0.689
***Viewing rate***	312.92 (151.4625–1401.485)^A^	350.88 (42.02–464.31)^A^	1495.58 (249.5–3658.89)^B^	0.028*
***VPI***	3.13 (1.515–13.9875)^A^	3.3 (0.42–4.3)^A^	14.88 (2.38–36.11)^B^	0.025 *
***Modified DISCERN***	1 (1–2)^A^	2 (2–2)^A^	3 (2–5)^B^	0.001*
***JAMA***	2 (2–3)	2 (2–4)	2 (2–4)	0.352

*GQS: Global Quality Score, JAMA: Journal of American Medical Association benchmark criteria

** Upper cases indicate significant differences between lines.

***Values are presented as median (first quartile-third quartile)

## Discussion

The present study represents an initial effort to examine the accuracy, quality, and reliability of audio/visual information available online regarding DME. The duration, source, number of views, comments, likes, dislikes, days since upload, II, VPI, and viewing rates for each video were recorded. The videos were categorized based on their content using the JAMA, GQS, and m-DISCERN criteria, and the information that they provided was evaluated in accordance with these classifications.

Due to YouTube has emerged as a prominent video-sharing platform without restraining, research indicated that dental students consider it ensuring the most valuable resources for complementary learning and frequently utilize for pragmatic purposes [26]. As mentioned earlier in this paper, YouTube videos on clinical dental techniques have become a popular resource for dental students and practitioners, but their quality and accuracy vary significantly. To the best of our knowledge, there is no available explanation of the efficacy of YouTube videos demonstrating DME clinical techniques or their accuracy in reflecting current best practices. YouTube videos depicting clinical techniques in other areas of medicine that reflect current best practices have shown mixed results, with overall quality being generally poor. Interestingly, a review of YouTube videos on oral hygiene practices during the COVID-19 pandemic found that only 6% were considered useful, with 40% being only slightly useful [27]. This suggests that while YouTube can be a valuable resource, the quality of content varies widely. Furthermore, during the aforementioned period, subsequent to declaration of COVID-19 as a pandemic, numerous academic institutions transitioned abruptly to fully online instruction [28]. Concurrently, students began independently seeking educational content online without adequate guidance from academic professionals. This transition may have necessitated the rapid and extemporaneous creation of online educational resources, including videos on popular social media platforms such as YouTube, to address the situation.

Studies across various medical specialties have found that the educational content of YouTube videos is often lacking for. In a review of surgical procedure videos, researchers reported a lack of comprehensive material across multiple surgical disciplines [29]. Similarly, a systematic review of YouTube videos on cardiopulmonary resuscitation revealed that the online information was of low quality, with contents often being shared by from unknown sources and with questionable accuracy [30]. It should be noted, however, that there were some contradictions in the findings. While the overall quality tended to be poor, videos uploaded by physicians and those focusing on surgical techniques generally scored higher in quality assessments. For instance, in a study on videos demonstrating the transoral endoscopic thyroidectomy vestibular approach, those uploaded by physicians had significantly higher quality scores [31]. However, these higher-quality videos often had lower popularity scores, indicating a disconnect between video quality and viewership. In fact, as another example, based on the research conducted by Goyal et al. [32], it was determined that a significant proportion of YouTube videos relating to carpal tunnel syndrome, namely 78%, included at least one statement that had the potential to cause confusion or misinterpretation. It can thus be concluded that while YouTube offers potential as an educational tool for dental clinical techniques for dental students, the accurate reflection of current best practices remains inconsistent. Accordingly, dental educators and professionals should play an active role in creating and curating high-quality, evidence-based content on YouTube to ensure that it accurately reflects current best practices in dentistry [27, 33]. To effectively harness YouTube’ s popularity and accessibility for educational purposes for dental students, there is a need for a peer-reviewed video library and a standardized approach to online medical education content [29, 34].

According to our results, 48% of the uploaded videos were of high quality. Although the majority of these videos were from dental companies, dental professionals, and health-related institutions, it was observed that the videos uploaded by specialists were generally of low quality. Similarly, it has been reported that videos from academic sources do not necessarily provide higher-quality information than those from non-academic sources [35]. This suggests that even videos from seemingly reputable sources may not accurately reflect current best practices. Nevertheless, while YouTube videos are often unreliable, those created by healthcare professionals tend to be more accurate. A study on men’s health topics on YouTube found that videos uploaded by physicians or healthcare organizations were usually more reliable [36]. However, these more reliable videos often had lower user engagement metrics compared to less reliable content, once again suggesting a disconnect between quality and popularity. Although the videos uploaded about DME can be easily understood by dentists or students, the insufficient information provided in the videos and the lack of sufficient information about loaders may raise concerns about reliability and accuracy. To achieve comprehensive educational videos for the purpose of effective dental student education, there exists a necessity for further enhancement of the videos uploaded by both academic/non-academic sources, which only a limited number of them adequately address all the requisite elements. In addition, the overall quality, accuracy, and reliability of the “educational” videos were quite high; however, while the quality of the “teaching technique” videos was also high, the reliability and accuracy were low. Similarly, a study examining YouTube videos related to dentistry found that videos classified under the “education” category had a higher degree of usefulness and informational value for laypersons, dental students, and dental professionals compared to those in broader search categories [33]. Furthermore, our results revealed that the accuracy of the “advertisement” videos was high, but both the quality and reliability were inadequate. Although the uploaders of these videos shared sufficient information about the originating channels, they did not pay attention to uploading quality content or sharing sufficient information. The reason for this may be that these videos serve the purpose of promoting products; thus, there are concerns about the potential biases that they may exhibit. Likewise, the quality and reliability of the videos in the “other” category were low, but the accuracy was of a medium level. Specifically, their content quality level was generally high, with an average rate of 48% (n = 27). In some studies, YouTube videos showed low quality content about their topic, which stands in contrast to our outcomes [37, 38]. We think that these differences were due to the different video uploaders, the educational status of the reviewers, and the fact that YouTube is an open access platform through which anyone can upload videos. In light of our research findings, it is essential that the internet be populated with high-quality videos containing comprehensive information about DME.

In the present study, when evaluating accordance to sources, a significant difference was observed only in terms of accuracy, and thus, the first null hypothesis was thus not fully supported by our data. Moreover, in our study, the authors discovered that videos uploaded by a YouTube channel had lower content quality; on the other hand, changes could be observed regarding their accuracy and reliability. The videos uploaded by dental companies were found to be more reliable, and videos posted by the health-related institutions were seen to be more accurate with a significant difference. Overall, videos uploaded by healthcare professionals, academic institutions, and official medical organizations tend to be more reliable and accurate than those from commercial sources or laypersons. Moreover, academic institutions bear the liability of directing students toward applicable and proper learning sources, including video-based lecture substances [39]. For instance, according to Kurt et al. [40], videos uploaded by public institutions, associations, or hospitals had significantly higher coronavirus index and total index scores compared to those uploaded by dentists. This suggests that health-related institutions provided more accurate information. Similarly, Rachmawati et al. [41] found that videos made by healthcare professionals scored higher in usefulness, GQS, and comprehensiveness compared to those by laypersons. However, there have been some contradictions in the literature. Videos from commercial companies had higher understandability and actionability scores but also contained more misinformation. Even though, the majority of the literature suggests that videos from healthcare professionals, academic institutions, and official medical organizations tend to be more reliable and accurate, the quality and reliability of online health information can still vary, and viewers should be cautious and selective when seeking information from platforms like YouTube [41–43]. Given these findings, consulting the source of the information on DME videos before viewing can enhance the accuracy of the content for students.

While in this study, the highest viewing rate was correlated with videos containing high-quality content, Altun et al. [44] reported that the highest score on this parameter was associated with intermediate content quality. Based on the results obtained here, even though there was no significant difference in video demographic data with respect to accuracy, there was a significant difference in the reliability and video duration. Similarly, when evaluated in terms of quality, it was observed that there was a significant difference with many video demographic data. Furthermore, when evaluated in terms of content, the second null hypothesis was not supported by our data, as there was a difference in quality, accuracy, and reliability.

The research revealed a positive correlation between the level of content quality and the reliability of videos, indicating that high-quality videos tend to be more reliable. Nevertheless, more accurate videos have also demonstrated greater reliability. At the same time, there was no correlation between the accuracy and quality of the videos. Therefore, the third null hypothesis was not fully supported by our data. Additionally, it was noted that videos with longer durations, more comments, and more views exhibited higher content quality. Moreover, while it was stated that videos with more views exhibited higher accuracy, videos with longer durations and more dislikes showed higher reliability. Longer video duration thus tends to be associated with higher quality and reliability scores. This is likely because longer videos allow for a more comprehensive and detailed information presentation [45, 46]. Interestingly, the number of dislikes can also be positively correlated with video quality. In another study, it was found that in the high-quality group, the number of dislikes was higher than in the low-quality group [47]. This counterintuitive finding might be explained by the fact that higher-quality videos often attract more viewers overall, leading to more engagement, including dislikes [45, 47]. Furthermore, a positive correlation was identified between the accuracy and content quality of videos with VPI/viewing rate. Likewise, a positive correlation was established between the number of likes and the scores on the GQS and the JAMA. Accordingly, it was observed that videos with a higher level of content quality and accuracy exhibited a positive correlation with a higher number of likes.

It is important to note that there are limitations to this study. First, the present study was limited to videos containing content in the English language. What this means is that there is a large number of DME videos that have not been assessed. Second, the study solely evaluated YouTube videos without examining the experiences of actual dental students, post-graduates, specialists, professionals, or patients. Another limitation of our study was analyzing only the first 100 videos on YouTube identified upon searching for our keywords. However, it is a known fact that most people do not read more than one or two pages of searching results they find on the internet [48]. Another limitation is that a three-day window was selected for the keywords. While it is possible that videos posted after the three-day period were not included, the selected timeframe was intended to capture a snapshot of the available content. This method can reduce the risk of selection bias by ensuring that the search was conducted uniformly across all keywords. To mitigate potential bias, future studies could consider extending the search period or conducting periodic searches over longer durations. This would help capture new videos and provide a more comprehensive dataset. This study also acknowledges the potential for selection bias due to the use of a single researcher and the influence of YouTube’ s algorithm, which is why three researchers evaluated the videos, and a new YouTube account was created. Nevertheless, future research should consider employing multiple researchers or automated video-sorting tools to enhance the objectivity and reliability of the video selection process. Studies have shown that these methods can significantly reduce bias and improve the quality of the selected content [49, 50]. We should also note the exclusion of YouTube videos longer than 30 minutes. While this criterion was set to ensure the manageability of the dataset and a focus on concise content, it may have inadvertently excluded some educational or in-depth videos that provided comprehensive information about DME.

Recognizing the dynamic nature of YouTube, with all of its videos, likes, dislikes, and views constantly changing, is crucial in a study such as this one. Despite the use of specific tools here, like the GQS, JAMA, and DISCERN scales, there is no consensus on a standardized method for evaluating healthcare-related videos. Consequently, the comprehensive evaluation of the videos remains subjective and depends on the discretion of individual researchers. Despite the constraints mentioned above, YouTube videos still offer immediate insights into current developments in DME. Conversely, future research could incorporate these aspects while assessing the efficacy of DME.

## Conclusion

As the number of videos on YouTube regarding DME is growing rapidly, it is essential to establish internationally recognized guidelines for producing high-quality videos. YouTube should require that profile information be added and confirmed during video uploading. For health-related videos, YouTube should require that uploaders only be from a healthcare institution, university, or healthcare-related facility. These regulations can make the YouTube platform more reliable and accurate. In this way, YouTube would be one step closer to becoming an educational tool that can be included in dentists’ clinical/preclinical education curricula. By refining the content through a more rigorous filtering process and using peer-reviewing procedures by healthcare professionals, the videos could provide more accurate and reliable information to the public. While YouTube serves as a potent medium for disseminating information to a broad audience, its potential can only be fully realized by promoting the ethical and qualified production and sharing of such videos.
